# Supplementary material for: Agaricus blazei-Based Mushroom Extract Supplementation to Birch Allergic Blood Donors: A Randomized Clinical Trial
Source: Nutrients. 2019 Oct 2;11(10):2339. doi: 10.3390/nu11102339 (PMC6836217; doi:10.3390/nu11102339)
Supplement: Supplementary file 1 [file nutrients-11-02339-s001.zip › Supplementary Material 1.pdf]

This site became the new ClinicalTrials.gov on June 19th. Learn more.

We will be updating this site in phases. This allows us to move faster and to deliver better services.

Show less

⚠ **IMPORTANT**: Listing of a study on this site does not reflect endorsement by the National Institutes of Health. Talk with a trusted healthcare professional before volunteering for a study. Read more...

ClinicalTrials.gov
A service of the U.S. National Institutes of Health

Saved Studies (1)

Give us feedback

Does Medicinal Mushroom Agaricus Blazei Protect Against Allergy and Asthma?

This study has been completed.

Sponsor:
Oslo University Hospital

Collaborators:
University of Oslo
ImmunoPharma AS

Information provided by (Responsible Party):
Lise Sofie Haug Nissen-Meyer, Oslo University Hospital

ClinicalTrials.gov Identifier:
NCT03198455

First received: March 15, 2017
Last updated: June 22, 2017
Last verified: June 2017
History of Changes

Purpose

Asthma and allergy is increasing in Norway and Western countries. Treatment is still mostly symptomatic. Extracts of the immunomodulatory and edible mushroom Agaricus blazei, such as Andosan™, have been shown to protect against asthma and allergy in murine models by changing the T helper cell 1(upregulation)-T helper cell 2 (downregulation) balance in the immune system. Andosan™ is produced in Japan and approved as food (mushroom juice) in Norway. Blood donors and possibly patients with pollen-derived allergy and asthma will be included in the

study.The aim is to examine whether Andosan™ i) has similar clinical effects against allergy and asthma in man as it has in mice, and ii) reduces drug use and increases frequency of blood donations. Blood donors or patients who are recruited with informed consent will be given Andosan™ or placebo orally as add-on treatment to ordinary treatment for 7 weeks during the Birch pollen season, and specific IgE will be measured before, during and after the intervention, in addition to basophil activation testing and filling out of a questionnaire.

Condition	Intervention
Pollen; Allergy, Asthma	Dietary Supplement: Agaricus blazei Murill-based mushroom extract, Andosan™<br>Other: Placebo

Study Type: Interventional
Study Design: Allocation: Randomized
Intervention Model: Parallel Assignment
Masking: Triple (Participant, Care Provider, Outcomes Assessor)
Primary Purpose: Prevention

Official Title: Does Medicinal Mushroom Agaricus Blazei Protect Against Allergy and Asthma?

Resource links provided by NLM:

MedlinePlus related topics: Allergy

U.S. FDA Resources

Further study details as provided by Lise Sofie Haug Nissen-Meyer, Oslo University Hospital:

Primary Outcome Measures:

- Questionnaire [Time Frame: Change from baseline at mid-season at approximately 3 months and after end of season at approx 6 months]

  Questionnaire about allergy and asthma symptoms and medication

Secondary Outcome Measures:

- Total IgE in serum [Time Frame: Change from baseline at approximately 3 months and 6 months]

  Total IgE (kU/l) for all allergies

- IgE anti-rBet v 1 in serum against birch pollen allergy [Time Frame: Change from baseline at approximately 3 months and 6 months]

  Specific IgE (kUA/l) to rBet v 1 allergen

- IgE anti-t3 in serum against birch pollen allergy [Time Frame: Change from baseline at approximately 3 months and 6 months]

  Specific IgE (kUA/l) to t3 Birch pollen extract

- Basophil Activation Test (BAT) [Time Frame: Change from baseline at approximately 3 months and 6 months]

  Basophil granulocytes are isolated from venous blood samples of study participants

- Cytokines in serum [Time Frame: Change from baseline at approximately 6 months]

  Th1, Th2, pro-and anti-inflammatory cytokines (pg/ml) in plasma was measured by Luminex multi cytokine kit analysis

Enrollment:	60
Actual Study Start Date:	February 2016
Study Completion Date:	October 2016
Primary Completion Date:	October 2016 (Final data collection date for primary outcome measure)

Arms	Assigned Interventions
Experimental: Andosan<br>The Agaricus blazei Murill-based mushroom extract, Andosan™, is given as one dosage 60 ml/day orally for 2 months. The intervention solution is given for 1 month's consumption at a time in a neutral plastic container	Dietary Supplement: Agaricus blazei Murill-based mushroom extract, Andosan™<br>Dietary Supplement: Agaricus blazei Murill-based mushroom extract, Andosan™
Placebo Comparator: Placebo<br>The placebo is drinking water with brownish food coloring, given as one dosage 60 ml/day orally for 2 months. The placebo solution is given for 1 month's consumption at a time in a neutral plastic container (same as for intervention/experimental solution).	Other: Placebo<br>Drinking water with food coloring and salt

Detailed Description:
Blood donors at Oslo University Hospital (OUH) Blood Bank with self-reported birch pollen allergy and/or asthma were recruited for the study during last 4 months of 2015 and first 2 months of 2016. They signed an informed consent form for the study and were randomized into Andosan™ and placebo Groups. The PI (MD) had the study key and the participants in the study were handled by other staff (nurses and bioengineers) at the blood bank. The participants were given a questionnaire that was filled out before and after the study. Before, during and after the study also blood samples were taken for allergy testing (IgE, BAT) or cytokine profiles. When the participants visited the blood bank after 3.5 weeks, they brought the study medicine vessel (plastic container) that was controlled for remaining study medicine (all should have been used) and given new study medicine for the remaining study period (3.5 weeks). The participants were

also asked about possible side effects of the study medicine. In the intervention arm, Agaricus blazei-based mushroom extract Andosan™, was given, which is produced as Health food in Japan and imported as food to Norway and provided for the study by Immunopharma company, Norway. Data were collected and analyzed together after the study. Statistical help was solicited from an OUH statistician.

Eligibility

Ages Eligible for Study:	Child, Adult, Senior
Sexes Eligible for Study:	All
Accepts Healthy Volunteers:	Yes

Criteria

Inclusion Criteria:

- Blood donors with birch pollen-derived allergy and asthma

Exclusion Criteria:

- Blood donors with other types of allergy and blood donors with Birch pollen allergy who are would not be in Southern Norway during most of the pollen season

Contacts and Locations

Choosing to participate in a study is an important personal decision. Talk with your doctor and family members or friends about deciding to join a study. To learn more about this study, you or your doctor may contact the study research staff using the Contacts provided below. For general information, see Learn About Clinical Studies.

No Contacts or Locations Provided

More Information

Responsible Party:	Lise Sofie Haug Nissen-Meyer, Project leader, Oslo University Hospital
ClinicalTrials.gov Identifier:	NCT03198455 History of Changes
Other Study ID Numbers:	2015/716
Study First Received:	March 15, 2017
Last Updated:	June 22, 2017

Individual Participant Data (IPD) Sharing Statement:
Plan to Share IPD: Undecided

Studies a U.S. FDA-regulated Drug Product: No
Studies a U.S. FDA-regulated Device Product: No

Additional relevant MeSH terms:

Asthma	Respiratory Hypersensitivity
Hypersensitivity	Hypersensitivity, Immediate
Rhinitis, Allergic, Seasonal	Immune System Diseases

Bronchial Diseases
Respiratory Tract Diseases
Lung Diseases, Obstructive
Lung Diseases
Rhinitis, Allergic
Rhinitis
Nose Diseases
Otorhinolaryngologic Diseases

ClinicalTrials.gov processed this record on September 15, 2017
